# Functional screening of aldehyde decarbonylases for long-chain alkane production by *Saccharomyces cerevisiae*

**DOI:** 10.1186/s12934-017-0683-z

**Published:** 2017-05-02

**Authors:** Min-Kyoung Kang, Yongjin J. Zhou, Nicolaas A. Buijs, Jens Nielsen

**Affiliations:** 1 Department of Biology and Biological Engineering, Chalmers University of Technology, Kemivägen 10, 412 96 Gothenburg, Sweden; 2 Novo Nordisk Foundation Center for Biosustainability, Chalmers University of Technology, 412 96 Gothenburg, Sweden; 3 Novo Nordisk Foundation Center for Biosustainability, Technical University of Denmark, Kogle allé, 2970 Hørsholm, Denmark; 4 Science for Life Laboratory, Royal Institute of Technology, 17121 Solna, Sweden; 5 Division of Biotechnology, Dalian Institute of Chemical Physics, Chinese Academy of Sciences, Dalian, 116023 China; 6 Evolva Biotech, Lersø Parkalle, 40-42, 2100 Copenhagen, Denmark

**Keywords:** Metabolic engineering, *Saccharomyces cerevisiae*, Alkane biosynthesis, Aldehyde decarbonylase, Biofuels

## Abstract

**Background:**

Low catalytic activities of pathway enzymes are often a limitation when using microbial based chemical production. Recent studies indicated that the enzyme activity of aldehyde decarbonylase (AD) is a critical bottleneck for alkane biosynthesis in *Saccharomyces cerevisiae*. We therefore performed functional screening to identify efficient ADs that can improve alkane production by *S. cerevisiae*.

**Results:**

A comparative study of ADs originated from a plant, insects, and cyanobacteria were conducted in *S. cerevisiae*. As a result, expression of aldehyde deformylating oxygenases (ADOs), which are cyanobacterial ADs, from *Synechococcus elongatus* and *Crocosphaera watsonii* converted fatty aldehydes to corresponding C_n−1_ alkanes and alkenes. The CwADO showed the highest alkane titer (0.13 mg/L/OD_600_) and the lowest fatty alcohol production (0.55 mg/L/OD_600_). However, no measurable alkanes and alkenes were detected in other AD expressed yeast strains. Dynamic expression of SeADO and CwADO under GAL promoters increased alkane production to 0.20 mg/L/OD_600_ and no fatty alcohols, with even number chain lengths from C8 to C14, were detected in the cells.

**Conclusions:**

We demonstrated in vivo enzyme activities of ADs by displaying profiles of alkanes and fatty alcohols in *S. cerevisiae*. Among the AD enzymes evaluated, cyanobacteria ADOs were found to be suitable for alkane biosynthesis in *S. cerevisiae*. This work will be helpful to decide an AD candidate for alkane biosynthesis in *S. cerevisiae* and it will provide useful information for further investigation of AD enzymes with improved activities.

**Electronic supplementary material:**

The online version of this article (doi:10.1186/s12934-017-0683-z) contains supplementary material, which is available to authorized users.

## Background

Global warming and depletion of fossil fuels are two urgent matters. Fossil fuels are finite energy resources, but the world energy demand has been increasing along with economic development and population growth. Moreover, increase in carbon dioxide emissions have caused the global temperature to rise resulting in dramatic environmental changes. Therefore, there has been growing interest in sustainable production of biofuels and bio-based chemicals using microorganisms, so called cell factories. Advances in metabolic engineering and synthetic biology enables the production of bio-based chemicals using microbial cell factories [1–5].

One of the most important microbial cell factories, *Saccharomyces cerevisiae* is generally recognized as safe (GRAS) and, it is an extremely well-characterized and tractable organism. Because of its robustness and tolerance towards various stress conditions, it has been intensively used to produce several advanced biofuels and chemicals [6–9].

Alkanes are indispensable chemicals in our daily lives. As major components of current petroleum fuels, the chain lengths of alkanes determine their applications, such as gas (C1–C4), gasoline (C4–C9), jet fuel (C8–C16), diesel (C10–18), and lubricants (C16–C30) [10]. In nature, a variety of organisms synthesize alkanes to protect them against threatening environmental conditions, or to sustain growth [11–13]. However, the alkane production level from natural producers is very low and the alkane formulas are not suitable to replace current petroleum-based alkanes [1, 2]. In addition, current alkane needs are only fulfilled after the challenging and costly cracking processes of crude petroleum. Therefore, many efforts have been made to engineer microorganisms to produce desirable types of alkanes. Several alkane biosynthetic routes have been discovered and various enzymes are available to synthesize alkanes in heterologous hosts [14, 15]. To date, three major precursors, fatty acyl-ACP (or CoA), fatty acids, and fatty aldehydes have been utilized to demonstrate alkane production in engineered microorganisms [14, 15]. Aldehyde decarbonylases (ADs), which were discovered in plants, insects, and cyanobacteria, can convert fatty aldehydes to the corresponding C_n−1_ alkanes by co-producing carbon monoxide (CO), carbon dioxide (CO_2_), or formate, respectively. In engineered microbial strains, expression of ADs from a plant (Arabidopsis CER1), an insect (*Drosophila melanogaster* CYP4G1), and various species of cyanobacteria (ADOs) displayed long-chain alkane products [12, 13, 16, 17]. However, the low enzyme activities of cyanobacteria ADs have been noticed and only allow for low alkane titers in *S. cerevisiae* [17–20]. To date, no direct comparative study of ADs from different origins for alkane biosynthesis has been carried out, so we performed a functional screening of different ADs to identify applicable enzyme candidates that can increase alkane production in *S. cerevisiae*. We constructed AD expressing yeast strains and presented the cell metabolite profiles of alkanes and fatty alcohols from each construct. In light of these results, we suggested the most efficient AD enzyme and proposed a strategy to enhance alkane production. As the mechanisms of AD enzymes are not clearly elucidated, our study explored to develop ideal AD enzymes for alkane biosynthesis in yeast cell factories. We anticipate the strategy described here will provide a feasible strategy to functional screening of other AD enzymes for various microbial cell factories.

## Results

### Construction of alkane biosynthetic pathways

**Fig. 1:**
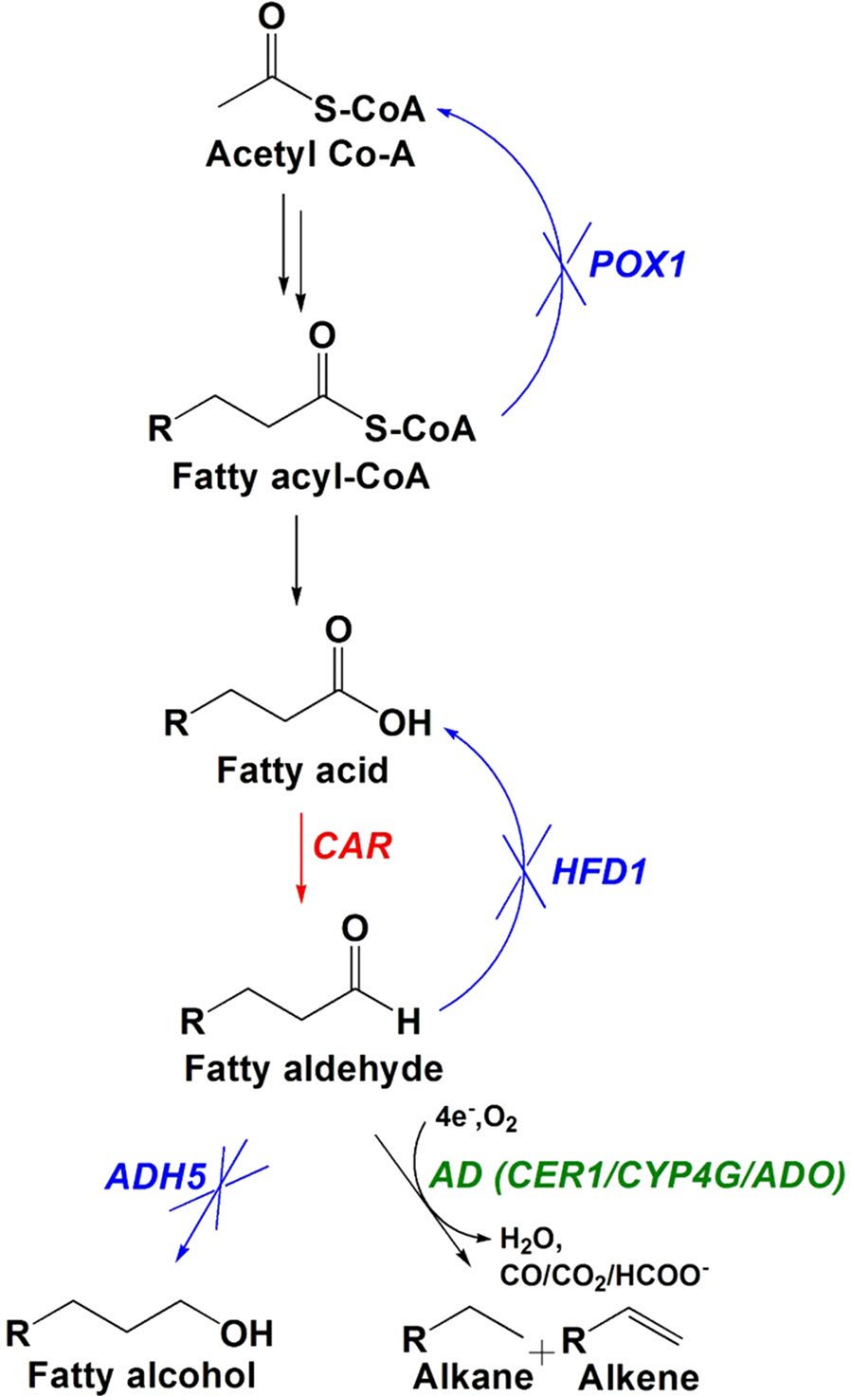
Scheme of alkane biosynthesis in engineered *S. cerevisiae* strains. The genes encoding fatty acyl-CoA oxidase, *POX1*, aldehyde dehydrogenase, *HFD1* and alcohol dehydrogenase, *ADH5*, were disrupted (*blue*) and alcohol dehydrogenase was overexpressed (*red*). ADs were inserted in an episomal plasmid and they were expressed to convert fatty aldehydes to alkanes (*green*)

In our previous study, the fatty acid biosynthetic pathway was engineered to supply sufficient fatty aldehydes in *S. cerevisiae* [18]. Here we used the engineered strain YJZ60 from this study as the background strain. The strain was optimized to accumulate fatty aldehydes in cells by deleting reversible reactions (*POX1* and *HFD1*) and expressing carboxylic acid reductase (*CAR*). One of the competing enzymes, alcohol dehydrogenase, Adh5, was deleted to reduce fatty alcohol accumulation (Fig. 1). In addition, the FNR/Fd reducing systems were expressed to supply sufficient electrons. Figure 1 and Table 1 summarizes information of YJZ60. To enable *S. cerevisiae* to convert the synthesized aldehydes to alkanes, we expressed various ADs by using the episomal plasmid pYX212 in the background strain YJZ60. We introduced three different types of ADs, the *ECERIFERUM1* (CER1) from Arabidopsis plant [16, 21], insect cytochrome p450s (CYP4G1 and CYP4G2) from *D. melanogaster* and house fly [12], and cyanobacteria aldehyde deformylating oxidases (ADOs) from *S. elongatus* [17, 18], *Crocosphaera watsonii*, *Thermosynechococcus elongatus*, and *Cyanothece* sp. PCC 7425 [22] (Table 1; Additional file 1: Figure S1). All AD candidates were selected by literature reviews and preliminary data. Codon-optimized ADO and CER1 genes were expressed under the control of the enhanced TDH3 promoter [23], while CYP4G1 and CYP4G2 were expressed under the control of the truncated HXT7 promoter, tHXT7p [24], yeast *S. cerevisiae* allowing constitutive expression independent of extracellular glucose levels. Additional file 1: Figure S1 provides brief features of the used gene expression modules. In the CSADO strain, *C. watsoni* and *S. elongatus* ADOs were co-expressed under the control of the *GAL1* and *GAL10* promoters, respectively (Table 1; Additional file 1: Figure S1) to alleviate the growth inhibition by separating cell growth and gene expression.

**Table 1:** Strains and plasmids used in this study

Name	Description	Reference
Plasmids		
pYX212	2 μm, AmpR, URA3, TPIp, pYX212t	R&D systems
pAlkane78	pYX212-(TPIp-Mdb5-FBA1t-CYC1t-MdCPR-TDH3p‐tHXT7P-CYP4G1‐pYX212t)	This study
pAlkane8	pYX212-(TPIp-Mdb5-FBA1t-CYC1t-MdCPR-TDH3p‐tHXT7P-CYP4G2‐pYX212t)	This study
pAlkane71	pYX212-(eTDH3p‐CER1-Syn27t-pYX212t)	This study
pAlkane67	pYX212-(eTDH3p‐SeADO‐pYX212t)	[17]
pAlkane83	pYX212-(eTDH3p‐CwADO‐pYX212t)	This study
pAlkane84	pYX212-(eTDH3p‐TeADO‐pYX212t)	This study
pAlkane85	pYX212-(eTDH3p‐CyADO‐pYX212t)	This study
pAlkane86	pYX212-(CYC1t-CwADO-Gal10p-Gal1p-SeADO-pYX212t)	This study
Strains		
DH5α	F^−^ (80d *lac*Z M15) (*lac*ZYA-*arg*F) U169 *hsd*R17(r^−^ m^+^) *rec*A1 *end*A1 *rel*A1 *deo*R96	
YJZ60	MATa MAL2-8c SUC2 his3Δ1ura3-52 hfd1Δpox1Δ Gal80Δ:: SeFNR + SeFd adh5Δ::(TPIp-MmCAR-FBA1t) + (PGK1p-EcFNR-CYC1t) + (TEF1p-EcFD-TDH2t) + (tHXT7p-npgA-ADH5t)	[17]
Con	YJZ60 strain harboring pYX212	This study
CYP4G1	YJZ60 strain harboring pAlkane78	This study
CYP4G2	YJZ60 strain harboring pAlkane8	This study
CER1	YJZ60 strain harboring pAlkane71	This study
SeADO	YJZ60 strain harboring pAlkane67	This study
CwADO	YJZ60 strain harboring pAlkane83	This study
TeADO	YJZ60 strain harboring pAlkane84	This study
CyADO	YJZ60 strain harboring pAlkane85	This study
CSADO	YJZ60 strain harboring pAlkane86	This study

### Evaluation of ADs for alkane biosynthesis in *S. cerevisiae*

**Fig. 2:**
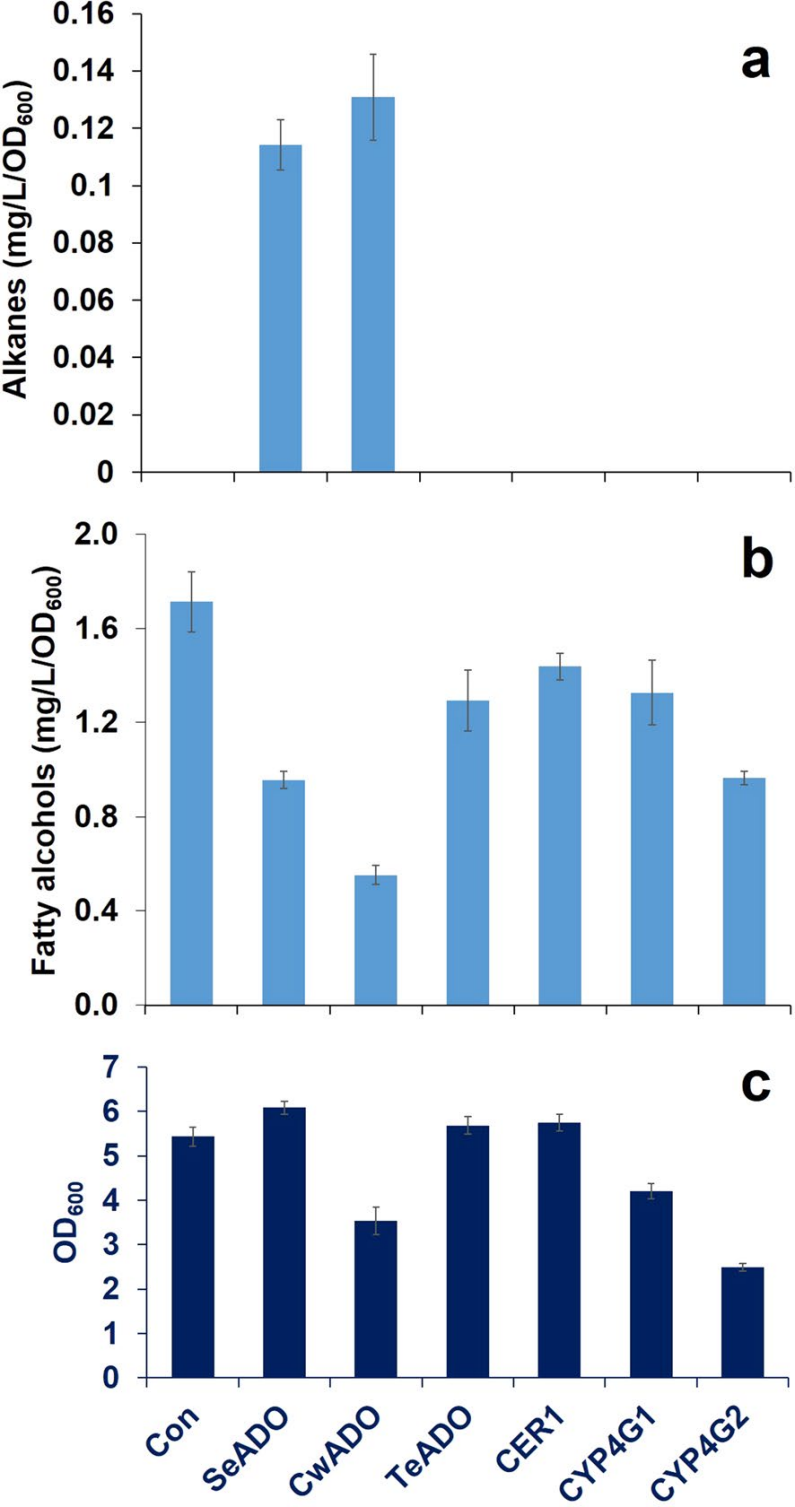
Comparison of alkane and fatty alcohol production by different AD expression in engineered *S. cerevisiae* strains. Alkane (**a**) and fatty alcohol (**b**) titers, and cell growth (**c**) were demonstrated from each engineered strain after 72 h culture in minimal media. All data represent the mean values and standard deviations from at least triplicate cultures

After the introduction of ADs in YJZ60, we carried out functional evaluation of three different types of ADs (CER1, CYP4G, and ADO). Among all the AD constructs tested, only two cyanobacterial ADOs from *S. elongatus* (SeADO) and *C. watsonii* (CwADO) produced long-chain alkanes and alkenes. Expression of ADOs from *S. elongatus* and *C. watsonii*, reached 0.11 and 0.13 mg/L/OD_600_ of total alkanes and alkenes, respectively, with different odd chain lengths from C11 to C17 (Fig. 2a; Additional file 1: Figure S2a). The major compounds in both strains were pentadecane (C15) and 7-pentadecene (C15:1) (Additional file 1: Figure S2a).

We found accumulation of fatty alcohols in all the engineered strains (Fig. 2b). This is consistent with previous observations that fatty alcohols are produced as significant by-products in engineered *S. cerevisiae* expressing alkane biosynthesis, and might be caused by endogenous aldehyde reductases (ALRs) and alcohol dehydrogenases (ADHs) [17, 18].The control strain Con without AD had the highest fatty alcohol accumulation (1.71 mg/L/OD_600_, Fig. 2) with even number chain lengths from C8 to C18 and the CwADO strain produced the lowest amount of fatty alcohols (0.55 mg/L/OD_600_) in the cells (Additional file 1: Figure S2). Other AD expressing strains produced fatty alcohol levels in between these strains, i.e. TeADO: 1.29 mg/L/OD_600_, CER1: 1.44 mg/L/OD_600_, CYP4G1: 1.33 mg/L/OD_600_, and CYP4G2: 0.97 mg/L/OD_600_ (Fig. 2b). The alkane production is much lower than the decrease in fatty alcohol accumulation when the CwADO and SeADO strains are compared with the control strain (Fig. 2a, b), and suggests that the functional ADs have a high binding affinity for fatty aldehydes, but low catalytic efficiency for alkane biosynthesis.

Though the CwADO strain had the highest alkane production and the lowest fatty alcohol production, this strain showed very poor growth (OD_600_ of 3.5 at 72 h) compared with the SeADO (OD_600_ of 6.1 at 72 h) and control strains (OD_600_ of 5.4 at 72 h), which might be attributed to toxicity (Fig. 2c). For this reason, the total amount of alkanes and alkenes produced by the CwADO strain (0.53 mg/L) is lower than with the SeADO strain (0.76 mg/L) (Fig. 2a). Improving cell growth of the CwADO expressing strain could therefore potentially further increase alkane production.

### Enhancement of alkane production

**Fig. 3:**
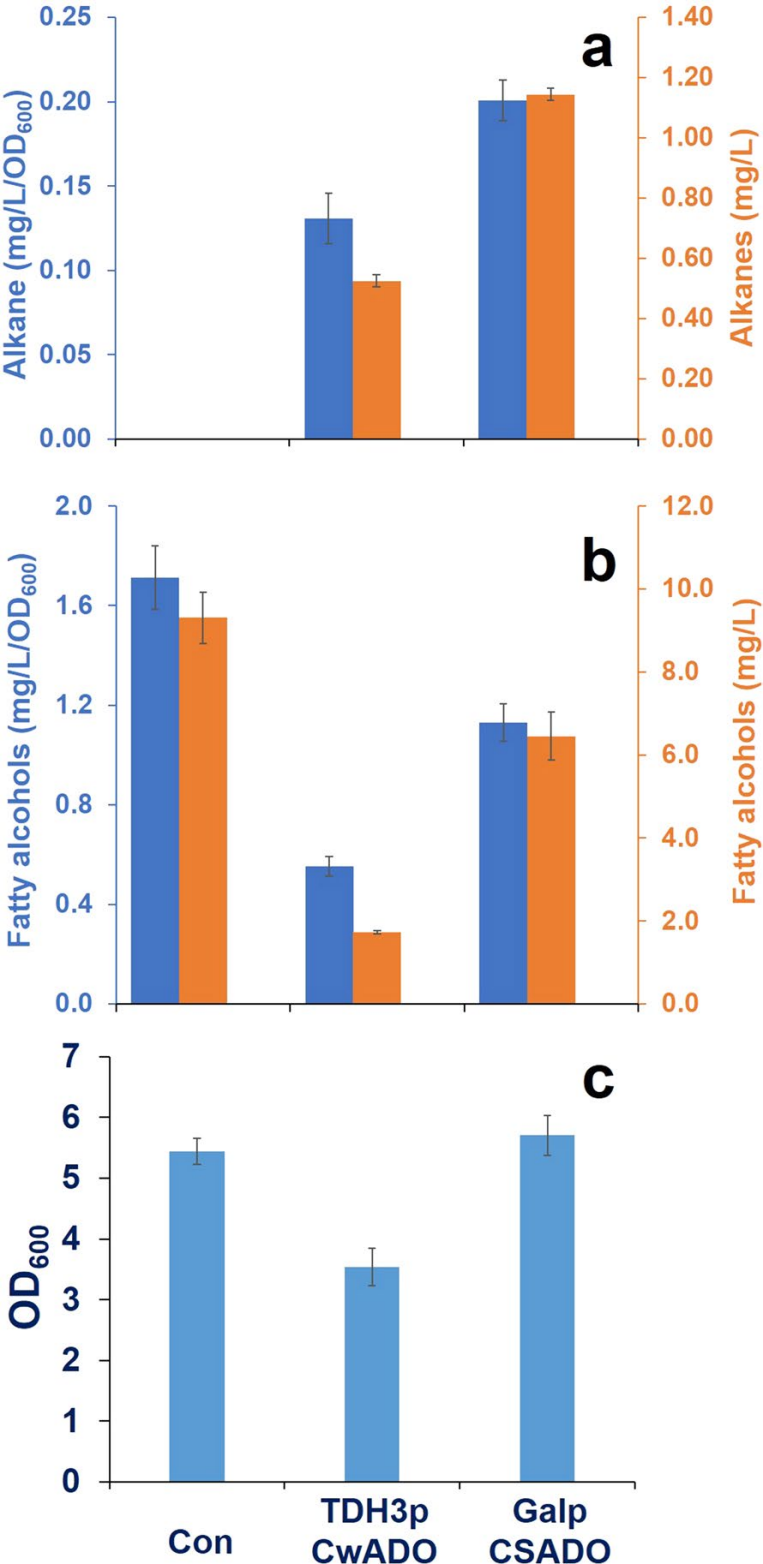
Enhancement of alkane production. Production of alkanes (**a**) and fatty alcohols (**b**), and two titer units (*left blue* mg/L/OD_600_, *right orange* mg/L) are used to display the level of metabolites. Cell growth of each strain is shown in (**c**). All data represent the mean values and standard deviations from at least triplicate cultures

In order to relieve the toxicity of expressing CwADO in the cell, we dynamically expressed CwADO by using the *GAL1* promoter (GAL1p) in combination with the *GAL80* deletion. It has been found that the *GAL1* promoter has very low expression in the glucose phase due to Mig1 repression, but is strongly expressed after glucose consumption in a *GAL80* deletion strain [25]. Hereby CwADO expression could be separated from cell growth, as has been previously applied for improving isoprenoid production by yeast [26]. To further increase alkane biosynthesis, we co-expressed SeADO under the control of the *GAL10* promoter (Gal10p). The resulting strain CSADO had significantly higher specific alkane production of 0.20 mg/L/OD_600_, (Fig. 3a) which was 35 and 45% higher compared with the CwADO and SeADO strains, respectively (Fig. 3a). We even detected undecane (C11) in the CSADO strain (Additional file 1: Figure S3a). Furthermore, CSADO had 62% higher biomass (OD_600_ of 5.7 at 72 h, Fig. 3c) than the strain CwADO expressed under the TDH3 promoter (Fig. 3c), which indicated that the dynamic control strategy relieved the toxicity of CwADO expression. As a benefit to improved cell growth, the alkane titer reached 1.14 mg/L, which is higher than with our previous strain A6 that had systematic pathway optimization [18]. This suggests that functional AD screening with dynamic expression could be an efficient strategy for enhancing alkane production in yeast.

## Discussion

In this study, long-chain alkane biosynthesis has been constructed via decarbonylation of fatty aldehydes by AD enzymes in *S. cerevisiae* [12, 16–18]. However, efficient incorporating of heterologous metabolic pathways into *S. cerevisiae* is challenging and strong endogenous ALRs/ADHs compete with the intermediate fatty aldehydes [18]. Indeed, low catalytic efficiency of ADs has been referred to as a critical bottleneck in alkane biosynthesis in engineered *S. cerevisiae* strains [17–19]. Therefore, it is worthwhile to screen efficient AD enzymes to provide a rationale enzyme for the improvement of alkane biosynthesis in microbial cell factories. To meet this goal, we carried out functional screening of ADs from different origins by comparing alkane and fatty alcohol accumulation in the cells. ADs were introduced using episomal plasmids and expressed in an engineered yeast strain, YJZ60, which provides fatty aldehydes as substrates for alkane biosynthesis. Of all the strains we tested, cyanobacteria ADOs (SeADO and CwADO) synthesized alkanes more efficiently than the CER1 and CYP4G enzymes (Fig. 2a). Even though very-long-chain (VLC) alkane production by CER1 and CYP4G1 have been reported in yeast strains [12, 16], we only found a reduction of fatty alcohol accumulation, but no detectable amounts of alkanes were produced in our yeast strains. We assume substrate preferences of plant and insect ADs might explain this. In fact, plants and insects synthesize VLC alkanes to form a wax layer and cuticular hydrocarbons, respectively, for environmental protection [27, 28]. Arabidopsis (CER1) synthesized VLC alkanes with the range of chain lengths being C27–C31 [16], and insect CYP4G family produces C23–C33 chain lengths VLC alkanes [12, 29, 30]. Through the distribution of fatty alcohol chain lengths (Additional file 1: Figure S2b), we predicted accumulation of fatty aldehydes with the even number of chain lengths, C8–C18 in our background strain, which might be unfavorable substrates for CER1 and CYP4Gs. Meanwhile, major alkane products synthesized by cyanobacteria ADOs are pentadecane (C15) and heptadecane (C17) [13], and both compounds were also major metabolites in our SeADO and CwADO yeast strains (Additional file 1: Figure S2a). Another presumable reason might be the environmental condition for proper function of the AD enzymes. The yeast cytosol may not be an optimized compartment for function of CER1 and CYP4Gs. To date, cyanobacteria ADO is the only group of AD enzymes, which have been demonstrated to have in vitro enzyme activity [31–33]. Plant origin CER1 is an endoplasmic reticulum membrane bound protein and CYP4G1 is localized in oenocytes [12]. The membrane protein expression often causes cell stresses and lower the biomass and expression. In addition, folding and solubility of eukaryotic membrane proteins is generally causing difficulties for performing kinetic studies [34], so no enzyme activity studies have been successfully conducted. Likewise, the membrane association in plant cells may cause problems for proper function of the enzyme in the yeast cytosol. Moreover, the relatively larger size of CER1 and CYP4Gs may cause problems with folding and expression. Moreover, alkane peaks in a GC–MS chromatogram cannot be detected if the AD enzyme has low and slow activity. Because inefficient aldehyde conversion to alkanes leads to high fatty alcohol formation, and the fatty alcohol peaks cover the alkane detection area further causing difficulties in detecting alkanes.

The CwADO enzyme was revealed as a better enzyme compared with the SeADO, but expression of CwADO caused poor growth and negatively affected the final titer of alkanes. Thus, we replaced the *TDH3* promoter with a *GAL1* promoter to control the gene expression, and we placed additional SeADO right after the *GAL10* promoter to co-express CwADO and SeADO in an episomal plasmid (Additional file 1: Figure S1). In our previous work, expression of additional ADO from *Nostoc punctiforme* (SeADO-NpADO) resulted in a 5% increase in alkane titer (0.82 mg/L) compared with only expressing SeADO (0.78 mg/L) [18]. In the case of the CSADO strain (CwADO-SeADO), co-expression of CwADO achieved a significant improvement in alkane titer by 33% (SeADO: 0.76 mg/L, CSADO: 1.14 mg/L) (Figs. 2a, 3a) and surprisingly no fatty alcohols with even number chain lengths C8–C14 were detected (Additional file 1: Figure S3b). In addition, the chain lengths of alkanes were extended from C11 to C17 (Additional file 1: Figure S3a) and growth was greatly improved in the CSADO strain (OD_600_ of 3.5 at 72 h, Fig. 3c) compared with the CwADO strain (OD_600_ of 5.7 at 72 h, Fig. 3c). Even though the CSADO strain lead to increase in alkane production, it was still far from the industrial requirements and even below the alkane titer in engineered *E. coli* (580.8 mg/L) and cyanobacteria (300 mg/L) [13, 35]. Unlike *E. coli* platforms, even the same enzymes involved in alkane biosynthesis produced much smaller quantities of alkanes in *S. cerevisiae* strains. Expression of CER1 enzyme in *E. coli* achieved the highest alkane titer [35], and the ADO enzymes from *T. elongatus*, and *Cyanothece* sp. also produced high amount of alkanes in *E. coli* [13, 22]. However, even trace alkanes were not observed in our CER1, TeADO, and CyADO yeast strains for uncertain reasons. Similar to the AD enzymes, expression of OleT decarboxylase, a terminal alkene producing enzyme, resulted in much higher terminal alkene production in *E. coli* (97.6 mg/L) than in *S. cerevisiae* (3.5 mg/L) [14]. To explain the big differences in alkane titer between *E. coli* and *S. cerevisiae*, other facts should be considered beyond the poor catalytic efficiencies of alkane producing enzymes.

## Conclusion

In this study, we examined the functional performance of ADs in engineered yeast strains. Based on the metabolite profiles of our engineered strains, we proposed advisable ADs and their applications to enhance alkane production in *S. cerevisiae*. Our study further provides a platform strain that can be used for screening ADs to be used for alkane production in yeast with the objective to develop a yeast cell factory that can be used for bio-based production of alkanes.

## Methods

### Construction of plasmids and yeast strains

Plasmids and strains used in this study are shown in Table 1. Plasmid construction was performed by the modular pathway engineering procedure as described by Zhou et al. [36]. DNA fragments for module construction were prepared by PCR amplification and each module was constructed by fusion PCR. PrimeSTAR was used for all the PCR processes, and primers used in this work were listed in Additional file 1: Table S1. Yeast transformation was conducted by LiAc/SS carrier DNA/PEG method [37], and constructed modules and linearized pYX212 plasmid backbone were used as DNA templates. To make yeast competent cells, the YJZ60 yeast strain was cultured at 30 °C and 200 rpm in YPD media, and transformants were selected on synthetic defined (SD) agar plates, which contained 6.9 g/L yeast nitrogen base without amino acids (Formedium, Hunstanton, UK), 0.77 g/L synthetic complete supplement mixture without uracil (Formedium), 20 g/L glucose (Merck Millipore) and 20 g/L agar (Merck Millipore). After the colony selection, yeast plasmids were extracted and introduced into *E. coli* DH5α competent cells to confirm the final plasmid constructs. *E. coli* colonies were selected on Lysogeny Broth (LB) agar plate containing 100 μg/mL ampicillin, and they were confirmed by DNA sequencing.

### Alkane biosynthesis and extraction

To produce alkanes, engineered *S. cerevisiae* strains were grown in 100 mL shake flasks containing 15 mL mineral media [38] plus 40 mg/L histidine and 30 g/L glucose at 30 °C and 200 rpm for 72 h. After the cultivation, 10 mL of cell cultures were harvested by centrifugation at 2000*g* for 10 min, and then cell pellets were dried for 48 h in a freeze-dryer. The dried cells were extracted by the method described by Khoomrung [39] by using 4 mL chloroform: methanol (v/v 2:1) solution containing hexadecane (0.5 µg/mL) and pentadecanol (0.01 mg/mL) as internal standards. After centrifugal vacuum concentration, the final dried samples were dissolved in 200 µL hexane.

### Metabolite analysis and quantification

Alkanes and alkenes were analyzed by gas chromatography (Focus GC, ThermoFisher Scientific) equipped with a Zebron ZB-5MS GUARDIAN capillary column (30 m × 0.25 mm × 0.25 mm, Phenomenex, Torrance, CA, USA) and a DSQII mass spectrometer (Thermo Fisher Scientific, Waltham, MA, USA). The GC program for alkanes and alkenes was as follows: initial temperature of 50 °C, hold for 5 min; then ramp to 140 °C at a rate of 10 °C per min and hold for 10 min; ramp to 310 °C at a rate of 15 °C per min and hold for 7 min. Fatty alcohols were quantitatively analyzed by GC-FID (Thermo Fisher Scientific, Waltham, MA, USA) equipped with a ZB-5MS GUARDIAN capillary column, and helium was used as carrier gas at a flow rate of 1 mL/min. GC program for fatty alcohol quantification was as follows: initial temperature of 45 °C hold for 2 min; then ramp to 220 °C at a rate of 20 °C per min and hold for 2 min; ramp to 300 °C at a rate of 20 °C per min and hold for 5 min.

## Additional file

**Additional file 1: Figure S1.** Scheme of plasmid constructs for alkane biosynthesis. pYX212 vector was used as a backbone to express ADs in engineered *S. cerevisiae* strains. **Figure S2.** Comparison of alkane and fatty alcohol production by different AD expression in engineered *S. cerevisiae* strains. Alkane (a) and fatty alcohol (b) titers were displayed with the information of chain-length distribution of each engineered strain. All data represent the mean values and standard deviations from at least triplicate cultures. **Figure S3.** Production of alkanes (a) and fatty alcohols (b) with the information of chain-length distribution in the CSADO strain. All data represent the mean values and standard deviations from at least triplicate cultures. **Table S1.** Primers used in this study.

## Abbreviations

AD: aldehyde decarbonylase
ADH: alcohol dehydrogenase
ADO: aldehyde deformylating oxygenase
ALR: aldehyde reductase
CAR: carboxylic acid reductase
PDH: pyruvate dehydrogenase
VLC: very-long-chain
