# Supplementary material for: Functional screening of aldehyde decarbonylases for long-chain alkane production by Saccharomyces cerevisiae
Source: Microb Cell Fact. 2017 May 2;16:74. doi: 10.1186/s12934-017-0683-z (PMC5414326; doi:10.1186/s12934-017-0683-z)
Supplement: Supplementary file 1 — Additional file 1: Figure S1. Scheme of plasmid constructs for alkane biosynthesis. pYX212 vector was used as a backbone to express ADs in engineered S. cerevisiae strains. Figure S2. Comparison of alkane and fatty alcohol production by different AD expression in engineered S. cerevisiae strains. Alkane (a) and fatty alcohol (b) titers were displayed with the information of chain-length distribution of each engineered strain. All data represent the mean values and standard deviations from at least triplicate cultures. Figure S3. Production of alkanes (a) and fatty alcohols (b) with the information of chain-length distribution in the CSADO strain. All data represent the mean values and standard deviations from at least triplicate cultures. Table S1. Primers used in this study. [file 12934_2017_683_MOESM1_ESM.pdf]

Additional file 1 for

Functional screening of aldehyde decarbonylases for long-chain alkane production in *Saccharomyces cerevisiae*

Min-Kyoung Kang, Yongjin J Zhou, Nicolaas A. Buijs, and Jens Nielsen *

*Correspondence to: Jens Nielsen E-mail: nielsenj@chalmers.se

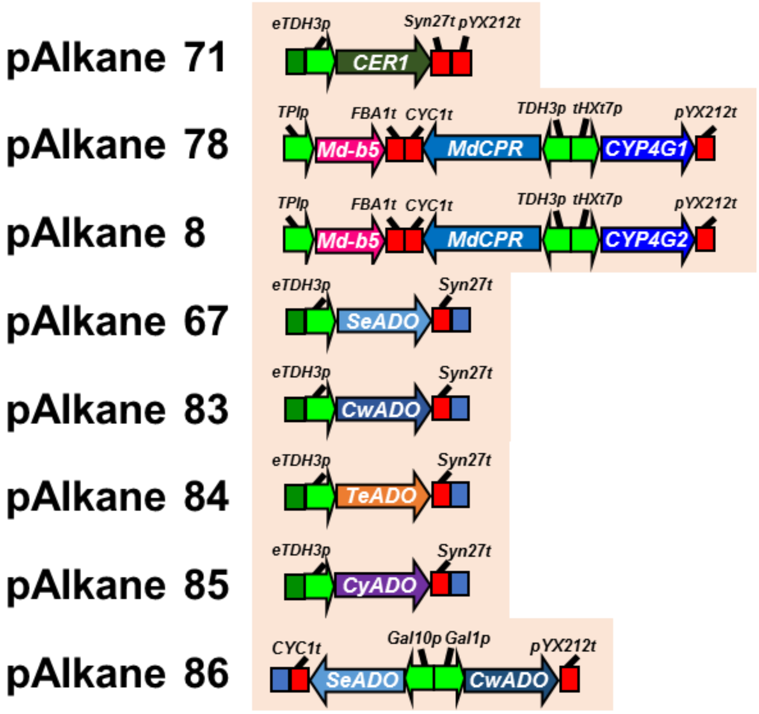

Figure S1. Scheme of plasmid constructs for alkane biosynthesis. pYX212 vector was used as a backbone to express ADs in engineered *S. cerevisiae* strains.

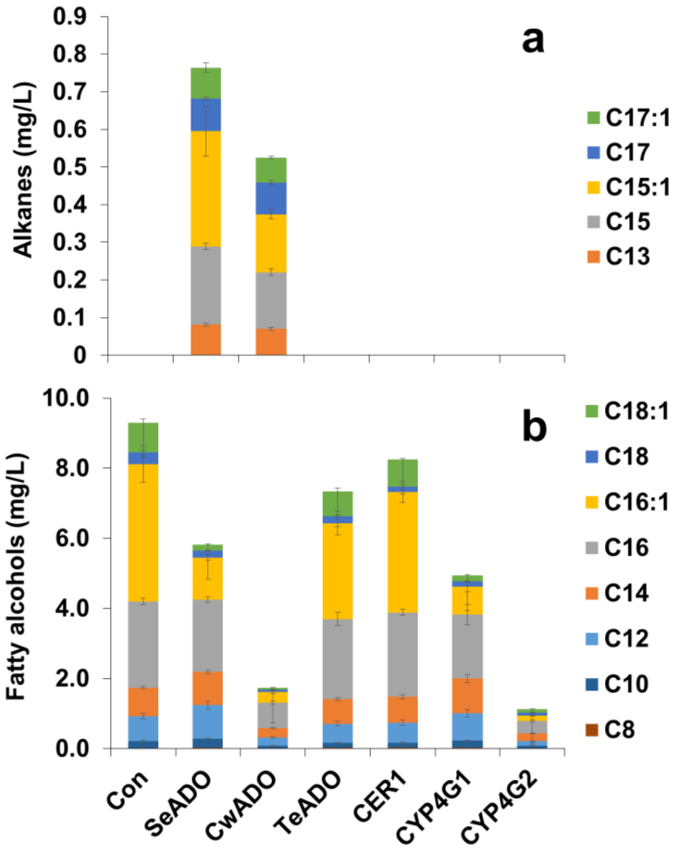

Figure S2. Comparison of alkane and fatty alcohol production by different AD expression in engineered *S. cerevisiae* strains. Alkane (a) and fatty alcohol (b) titers were displayed with the information of chain-length distribution of each engineered strain. All data represent the mean values and standard deviations from at least triplicate cultures.

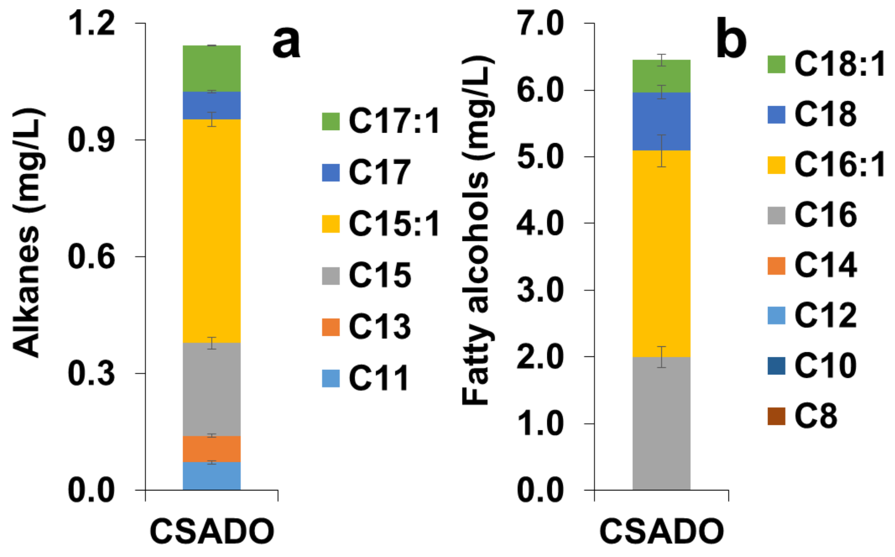

Figure S3. Production of alkanes (a) and fatty alcohols (b) with the information of chain-length distribution in the CSADO strain. All data represent the mean values and standard deviations from at least triplicate cultures.

Table S1. Primers used in this study

Name	Sequence (5’→ 3’)
pYX212-F	TAGGGCCCACAAGCTTACGCGTCGACCCGGGTATCC
pYX212-R	GCCGTAAACCACTAAATCGGAACCCTAAAGG
eTDH3p-F	CTTTCACCAGCGTTTC
TDH3p-F	CTCGAGTTTATCATTATCAATACTGCCATTTC
TDH3p-R	GTTTGTTTATGTGTGTTTATTCGAAACTAAGTTCTTGGTG
tHXT7p-F	GTATTCTTTGAAATGGCAGTATTGATAATGATAAACTCGAGCTCGTAGGAACAATTTC G
tHXT7p-R	CATTTTTTGATTAAAATTAAAAAAACTTTTTGTTTTTGTG
TDIp-F	GTTTAAAGATTACGGATATTTAACTTACTTAGAATAATG
TDIp-R	CATTTTTAGTTTATGTATGTGTTTTTTGTAG
GAL1p-R	CATTATAGTTTTTTCTCCTTGACGTTAAAG
GAL10p-R	CATTTATATTGAATTTTCAAAAATTCTTAC
FBA1t-F	GTTAATTCAAATTAATTGATATAGTTTTTTAATGAG
FBA1t-R	AGTAAGCTACTATGAAAGACTTTACAAAGAAC
CER1-F	CAAGAACTTAGTTTCGAATAAACACACATAAACAAACAAAATGGCTACAAAACCAGG TGTCTTG
CER1-R	CTAGACAGTTATATATATATATATATATATATATATACCACCCATTAGTGATGAGGTAACA ACAATGG
CYP4G1-F	CAAAAACAAAAAGTTTTTTTAATTTTAATCAAAAAATGGCCGTTGAAGTTGTTCAAGAA ACC
CYP4G1-R	GGATACCCGGGTCGACGCGTAAGCTTGTGGGCCCTATTAAGCAACAGTAGCGTATT GTCTC
CYP4G2-F	CACAAAAACAAAAAGTTTTTTTAATTTTAATCAAAAAATGGACTCCGCCAACAACTC
CYP4G2-R	GGATACCCGGGTCGACGCGTAAGCTTGTGGGCCCTATTACATTGCCTTCATTGCTT C
MdCPR-F	CAAGAACTTAGTTTCGAATAAACACACATAAACAAACAAAATGAGTGCCGAACACGT TGAAG
MdCPR-R	GTGACATAACTAATTACATGACTCGAGGTCGACGGTATCTTAACTCCAAACATCAGC GGAG
Md-b5-F	CTATAACTACAAAAAACACATACATAAACTAAAAATGTCCTCCGAAGATGTAAAG
Md-b5-R	CTCATTAAAAAACTATATCAATTAATTTGAATTAACTTATTGTGACTTAGTACCAAAG
SeADO-F	GAACTTAGTTTCGAATAAACACACATAAACAAACAAAATGCCACAATTAGAAGCCTCC
SeADO-R	GATACCCGGGTCGACGCGTAAGCTTTGAAAGATGATACTCTTTATTTCTAGACAGTT ATATATATATATATATATATATATACCACCCATTAGACTGCTGCCAAACCGTATGC
CwADO-F	GAACTTAGTTTCGAATAAACACACATAAACAAACAAAATGCAAGAATTGGCAGTCAG AAGTG

CwADO-R	CTAGACAGTTATATATATATATATATATATATATACCACCCATTAGACAGCGGCTAAA CCGTGGGCTG
TeADO-F	GAACTTAGTTTCGAATAAACACACATAAACAAACAAAATGACTACTGCCACCGCTACT CCTG
TeADO-R	CTAGACAGTTATATATATATATATATATATATATACCACCCATTAAGCACCAGTCAAAC CATAAAC
CyADO-F	GAACTTAGTTTCGAATAAACACACATAAACAAACAAAATGCCACAAGTTCAATCCCCA TCC
CyADO-R	CAGTTATATATATATATATATATATACCACCCATTAAGCAGCGGACAAACCATA AACAG
CwADO-F(86)	GTTAATATACCTCTATACTTTAACGTCAAGGAGAAAAAACTATAATGCAAGAATTGGC AGTCAGAAGTG
CwADO-R(86)	GGATACCCGGGTCGACGCGTAAGCTTGTGGGCCCTATTAGACAGCGGCTAAACCG TGGGCTG
SeADO-F(86)	GTCCATCCAAAAAAAAAGTAAGAATTTTTGAAAATTCAATATAAATGCCACAATTAGA AGCCTCCTTAG
SeADO-R(86)	CATAACTAATTACATGACTCGAGGTCGACGGTATCTTAGACTGCTGCCAAACCGTAT GCGGAC
